# Dietary Habits and Oral Hygiene as Determinants of the Incidence and Intensity of Dental Caries—A Pilot Study

**DOI:** 10.3390/nu15224833

**Published:** 2023-11-19

**Authors:** Dominika Mazurkiewicz, Malwina Pustułka, Jagoda Ambrozik-Haba, Maciej Bienkiewicz

**Affiliations:** The Faculty of Biotechnology and Food Science, Wrocław University of Environmental and Life Sciences 25 Norwida St., 50-375 Wrocław, Poland; 114861@student.upwr.edu.pl (M.P.); jagoda.ambrozik-haba@upwr.edu.pl (J.A.-H.); maciej.bienkiewicz@upwr.edu.pl (M.B.)

**Keywords:** dental caries, diet, oral hygiene, nutrition, dietary habits

## Abstract

Objectives: The aim of this study was to assess the influence of dietary and hygiene habits on the prevalence and intensity of dental caries. A group of 148 adults participated in the study. Methods: A self-administered nutrition and oral hygiene questionnaire was used to assess dietary habits and oral hygiene routine. A preventive diet index (PDI), a cariogenic diet index (CDI), and an oral health hygiene and prevention index (OHHPI) were created based on part of the questions. The intensity of dental caries among the respondents was investigated by the decayed, missing, and filled teeth index (DMFT), which was estimated on the basis of data obtained during the dental examination. Results: The study showed that 97% of the respondents had filled carious cavities, while untreated carious cavities were observed in up to 78% of the study participants. The respondents had an average consumption of preventive products and a low consumption of caries-stimulating foods. The preventive dietary index (PDI) value was higher in the female group than in the male group. A more frequent consumption of caries-inhibiting products was demonstrated by those declaring that they took various types of dietary supplements. The use of health-promoting sugar substitutes by the respondents was associated with a lower intensity of dental caries and a more frequent consumption of preventive products. Conclusions: The analysis of the survey results indicates the need to implement educational activities aimed at increasing public awareness of the prevalence of dental caries among adults.

## 1. Introduction

Tooth decay is a disease of civilisation that occurs in all age groups. It is estimated that nearly 2.4 billion people have or have had carious lesions. Moreover, caries is the most common cause of tooth loss among adults [1]. The World Health Organisation (WHO) defines dental caries as a pathological, localised process of exogenous origin that leads to decalcification of the enamel, the breakdown of hard tissues, and cavity formation [2]. Dental caries is a disease caused by the interaction of environmental, behavioural, and genetic factors. These factors include the substrate, easily fermentable dietary carbohydrates, which are metabolised by carious bacteria in the oral cavity, thereby stimulating plaque growth. Individual factors such as the quality of the tooth structure, the amount of saliva secreted, and the time at which plaque acts on the enamel affect the development of the disease [3]. Dental plaque is a natural form of colonisation of tooth enamel by oral bacteria. Plaque occurs in both individuals without symptoms of caries and those with diagnosed carious cavities. Crucial in the development of caries pathogenesis is the composition of the plaque-forming microbiota [4].

### 1.1. Influence of Nutrition

The main determinant of the occurrence of the carious process is the supply of sugars in the diet. According to the recommendations of the World Health Organisation, the intake of simple sugars during the day should not exceed 10% of the total energy requirements [5]. Research indicates that the greatest threats to oral health are high-sugar snacks and drinks consumed between main meals, as they provide the substrate necessary for the caries process to occur. Another important aspect is the regularity of the meals consumed throughout the day [6]. If the intervals between meals are too short, they interfere with the anti-cariogenic action of saliva [7]. Therefore, according to healthy eating recommendations, the time between meals should be from 3 to 4 h. Healthy individuals should also refrain from snacking at night, due to reduced saliva production during this part of the day. In order to prevent or treat caries, the diet should be modified so that products of a preventive nature prevail over those that induce the caries process [8].

### 1.2. Cariogenic Foods

Oral health is largely determined by the amount and frequency of cariogenic substances supplied with the diet. Caries-inducing substances include easily fermentable carbohydrates, particularly non-dairy free sugars, which are the substrate for the metabolic processes of carious bacteria, the products of which are acids responsible for the demineralisation of enamel. These sugars include sucrose, glucose, and fructose, which are found in large quantities in all kinds of sweets, sugar, honey, syrups, sweetened drinks, most often carbonated drinks, and fruit juices [6,9]. Gelatinised starch, i.e., starch that has undergone heat treatment, is also a food ingredient that induces cariogenesis [10]. Enamel demineralisation occurs when the pH of plaque drops to around 5.5. Acidic products such as citrus fruits, fermented or pickled vegetables, vinegar, vinaigrette-type dressing, and sweetened beverages, which include malic, citric, carbonic, and phosphoric acid as acidity regulators, and most of which have a pH value of less than 4, are responsible for lowering the pH in the mouth [11]. Another predisposing factor for the development of a carious cavity are foods with a sticky or mushy texture. Toffee-type sweets, fudge, crackers, or crisps have a high saccharide content, and their texture is conducive to sticking to the tooth surface. This combination creates optimal conditions for caries development [6,10].

### 1.3. Foods of a Preventive Nature

There are also foods that, due to their chemical composition and effects on the oral environment, can effectively protect the dentition against the development of pathological processes [12,13]. Cow milk proteins reduce the adhesion of cariogenic bacteria to hydroxyapatite, thus reducing the ability of the bacteria to metabolise sugars, resulting in a decrease in plaque mass. Lactose is fermented more slowly than sucrose, meaning that the pH of the oral cavity does not drop to optimal values for the development of the carious process. Calcium and phosphate, supplied with milk, contribute to remineralisation by saturating the enamel and saliva with these mineral ions. Milk and its unsweetened products are highly recommended for the prevention of dental caries [6,13,14]. A particularly important group of products whose primary ingredient is milk are hard cheeses (parmesan, pecorino, and grana padano). Their consumption is followed by the formation of a phosphopeptide–casein complex with calcium phosphate, which provides a reservoir of calcium ions for the enamel mineralisation and acts on plaque by raising the pH value [6,13,15]. Arginine-rich foods (nuts, soya, tuna, and some vegetables) have similar, rapid pH-raising properties. Arginine in saliva is broken down by plaque bacteria into alkali, thereby neutralising acids that are the direct cause of tooth decay. Additionally, changes in the pH of the oral environment result in an increase in the levels of Streptococcus sanguinis and Streptococcus gordonii, which are associated with protection against pathological processes, at the expense of the bacteria that induce these processes [16,17,18]. Among the many properties of fibre affecting the human body, a not insignificant one is the prevention of the development of the caries process by slowing glucose absorption and enhancing saliva production [8,12,13]. A multidirectional mechanism of caries reduction by compounds from the polyphenol group, which occur in vegetables, fruits, coffee, and tea, has been confirmed in studies. These bioactive compounds inhibit bacterial glucotransferase activity and reduce the ability of the bacteria to adhere, which limits the growth of cariogenic bacteria and the production of organic acids. In addition, polyphenols impair salivary amylase activity, making it more difficult for the oral microbiota to access glucose [6,19,20].

### 1.4. Other Pillars of Caries Prevention

In addition to diet, as discussed in the previous chapter, caries prevention consists of proper oral hygiene, meaning regular cleaning of the teeth, the spaces between them, and also the tongue, in addition to the supply, most often via oral hygiene products, of fluoride compounds, which are responsible for mineralisation of the hard tissues of the human body [21]. It is recommended to brush the teeth at least twice a day. After the second brushing, one should refrain from eating and drinking, with the exception of mineral water, due to the reduced natural cleansing process of the oral cavity by the tongue and facial muscles and reduced saliva production during the night. Too frequent brushing, improper technique, and an incorrect softness of the bristles of the toothbrush can contribute to damage to the enamel or gums. Proper tooth brushing should last approximately 2 min, as this length of time ensures that all surfaces of each tooth are cleaned and the action of fluoride ions is activated [22]. In addition to brushing, it is worthwhile to include additional cleaning of interdental spaces that may be missed by brushing in the daily oral hygiene process. This can be achieved with dental floss, an interdental brush, or an irrigator [23,24,25]. Additionally, oral rinses can be used to extend caries prevention. These rinses contain, among other ingredients, chlorhexidine and fluoride in varying concentrations. Chlorhexidine has antibacterial properties, which consist of its ability to damage the wall of Gram-positive bacteria, which include the Streptococcus mutans species. The presence of fluoride ensures the maintenance of its appropriate concentration in the saliva and on the dental plaque, which affects the quality of the hard tissue and limits the conditions for the growth of harmful bacteria [21,26,27,28,29,30,31,32].

## 2. Materials and Methods

### 2.1. Study Group

Recruitment of volunteers for this study took place in a dental office. The eligibility criterion for taking part in the study was signing informed and voluntary consent to participate in the study. Each participant was provided with information about the course of the study, opportunities to ask questions, or the option to withdraw from the study at each stage.

The study was conducted from August 2022 to March 2023, and the research group consisted of 148 people aged 18 and above. Approval for the study was obtained from the Rector’s Commission for Research Ethics operating at the Wrocław University of Life Sciences, number 14/2023.

The characteristics of the studied group, including its socio-demographic characteristics, are presented in Table 1.

### 2.2. Survey Questionnaire

A self-administered nutrition and hygiene questionnaire was developed to assess dietary and hygiene habits. The questionnaire consisted of four parts. The first included questions allowing for the determination of the general sociodemographic characteristics of the participants. The following sections contained questions based on which an analysis of dietary habits was carried out and oral health hygiene and prevention was investigated. In the course of the study, an attempt was also made to estimate the decayed, missing, and filled teeth index (DMFT) on the basis of a dental review during a dental office visit.

### 2.3. Preventive and Cariogenic Diet Index

The preventive diet index (PDI) consisted of six questions assessing the frequency of consumption of products with the potential to reduce the development of the caries process. The cariogenic diet index (CDI) consisted of six questions assessing the frequency of consumption of products that potentially induce the formation and development of dental caries.

The questions contained in the validated Kom-PAN questionnaire (questionnaire for the study of dietary views and habits and procedure for data processing—“KomPAN”—developed by the Behavioural Determinants of Nutrition Team, Committee for Human Nutrition Science, Polish Academy of Sciences) were used to create the indices [33,34]. Table 2 shows the questions used as the basis for calculating the preventive diet index (PDI) and the cariogenic diet index (CDI).

The resulting data had a qualitative data format, and the questions were ‘question-scales’ with an increasing frequency of consumption from ‘never’ to ‘several times a day’. The transformed categories could be analysed in the same way as real numerical data, but such data do not have a distribution that follows a normal distribution. This required the use of non-parametric tests in the statistical analysis or prior logarithmisation of the data [34].

In order to standardise the way the results were compiled and interpreted, it was recommended to use ranks and/or daily frequency indices expressed as multiples/day. The method of converting the qualitative data into scores is presented in Table 3 [34].

The frequency of consumption corresponded to the score awarded in the indexes, which was then summed. The point totals were subjected to a breakdown according to a three-point scale that indicated the level of frequency of consumption of the food groups studied. The classification rules for the scores are shown in Table 4.

### 2.4. Oral Health Hygiene and Prevention Index (OHHPI)

The oral health hygiene and prevention index (OHHPI) is a self-reported tool that assesses the quality of respondents’ oral hygiene maintenance and prevention knowledge. The selection of questions forming the foundation for the OHHPI was based on a literature review. According to the American Dental Association (ADA), for good oral health hygiene and prevention, teeth should be brushed twice a day with fluoride-containing toothpaste, and interdental spaces should be cleaned daily. The ADA also recommends chewing gum as a preventive measure. A healthy diet, limiting the intake of cariogenic foods, and regular visits to the dental office are also important recommendations [35]. The above guidelines correspond to those in place in Poland [36,37]. Table 5 shows the questions included in the index, the possible answers, and the score that was assigned to them.

All scores for the given answers were then summed and divided according to a three-point scale (as in the calculation of the PDI and CDI indices) (Table 5).

### 2.5. The Decayed, Missing, and Filled Teeth index (DMFT)

As part of the dental visit, each participant in the study had a dental check-up, which provided the information necessary to calculate the DMFT index. The decayed, missing, and filled teeth index (DMFT) provides information on the number of teeth affected by caries in a given population or individual. It is the ratio of teeth affected by caries (D), teeth extracted due to caries (M), and teeth with filled carious cavities (F) to the number of all individuals surveyed (T). The index can take values from 0 to 28 or from 0 to 32 [38,39].

### 2.6. Statistical Analysis

The normality of the distribution of the results obtained was tested by using the Shapiro–Wilk test. Descriptive statistics were used to determine the median (Me) and lower and upper quartiles (Q1:Q3). The variables did not follow a normal distribution, so non-parametric tests were used in a further analysis. The Kruskal–Wallis test and Mann–Whitney U test were used to determine differences between the independent groups. Spearman’s correlation coefficient and Kendall’s tau were used to examine correlations between the variables. A *p*-value ≤ 0.05 was interpreted as statistically significant. All statistical analyses were performed using STATISTICA (version 13.1 PL; StatSoft Inc., Tulsa, OK, USA; StatSoft, Kraków, Poland).

## 3. Results

### 3.1. Socio-Demographic Factors and BMI

Table 6 shows the influence of selected socio-demographic factors and BMI on the PDI, CDI, OHHPI, and DMFT values.

This study attempted to assess the influence of gender, age, place of residence, educational level, occupational status, and BMI values on the frequency of consumption of potentially caries-reducing and caries-inducing foods, the level of oral health hygiene and prevention, and the caries index. The course of the study showed that the study participants declared a low or medium level of consumption of preventive products in the context of dental caries.

Considering the age distribution of the study group, the highest level of consumption of preventive products was characterised by those in the age range 35–59 years (Me = 4.17), and the lowest was characterised by the oldest study participants, whose age ranged from 60–75 years (Me = 4.14). Age did not have a statistically significant effect on the value of the preventive diet index (*p* = 0.510) (Table 6).

The next variable tested was the BMI value. This variable was not statistically significant (*p* = 0.472). Obese (14.19% of the group) and normal-weight (50.68% of the group) subjects reported medium levels of consumption of products with preventive potential, and overweight subjects (34.46% of the group) reported low levels of frequency of consumption of products with preventive potential. Obese subjects by BMI had the highest median value (Me = 4.70) of the preventive diet index, while overweight subjects had the lowest median value (Me = 3.78) of the preventive diet index.

Place of residence was not a statistically significant variable (*p* = 0.712) in terms of preventive dietary index values. However, it is noteworthy that residents of larger cities (6.76% of the group) showed a more frequent consumption of preventive products compared to residents of cities with smaller populations (75% of the group) and of rural areas (16.89% of the group).

The group of people with primary and vocational education had the highest value of the preventive diet index (Me = 4.62); the next group in terms of the frequency of consumption of preventive products were those with tertiary education (Me = 4.06), while those with secondary education had the lowest value of the index (Me = 3.74). Education did not significantly affect the PDI index value (*p* = 0.353).

The unemployed had the highest preventive dietary index value (Me = 5.17), followed by students (Me = 4.12) and employees (Me = 4.11), and the lowest index value was found among those who were retired or receiving a pension (Me = 4.07).

The study showed that gender had a statistically significant effect on the preventive diet index value (*p* = 0.007). The median PDI in the women’s group was 4.48, while in the men’s group it was 3.21. It was shown that women were more likely to consume products that can reduce the development of tooth decay.

The cariogenic diet index represented the frequency of consumption of products that potentially stimulate the development of dental caries. Taking into account the age distribution of the study group, the youngest participants were characterised by the most frequent consumption of products negatively affecting oral health (Me = 1.27). Men were more likely to consume products harmful to the teeth (Me = 1.36) compared to women (Me = 1.21). The cariogenic diet index reached the highest values in the group with a normal BMI (Me = 1.72). The group most likely to consume cariogenic foods were residents of large cities (Me = 1.48). On the basis of the analysis of the results obtained, it was shown that the lowest CDI values were obtained in the group of people with primary education and in the people in the pension/retired group (Me = 0.81 and Me = 1.20, respectively) (Table 6).

Using the oral health hygiene and prevention index, the quality of hygiene procedures performed by the respondents was assessed, and their knowledge of fluoride prophylaxis and the effect of chewing gum on oral health was tested.

The highest index value was observed in the 35–59 age group (Me = 5.03). Both the youngest and oldest study participants had lower OHHPI values, with a median value of 4.50 in both cases.

The OHHPI values by gender, place of residence, education, employment status, and BMI were similar. Women had a slightly higher index value than men (5.20 and 5.00, respectively). BMI had no effect on the value of the oral hygiene and prevention index. The median for each group was 5.00. Those living in large cities had the lowest hygiene index value (Me = 4.31) compared to the study participants living in smaller towns and villages (5.00). Participants with secondary and tertiary education performed best in terms of OHHPI values (both groups had an OHHPI value of 5.00).

However, it is noteworthy that none of the study variables had a statistically significant effect on the CDI and OHHPI index values (Table 6).

When assessing the dental caries index, no significant correlations were observed between its value and the gender and place of residence of the study participants. A significant effect on the DMFT value was observed for age (*p* < 0.01). The number of teeth affected by caries and its sequelae increased with age. The lowest value of the index was obtained for respondents aged 18–34 years (Me = 7.00) and the highest was obtained for those aged 60–75 years (Me = 10.00).

BMI also had a statistically significant effect on the DMFT index (*p* < 0.01). The lowest value of the dental caries index was observed in the group of subjects with a normal BMI (Me = 7.00). The value of the index increased with increasing BMI values. The magnitudes determined for respondents with normal weight (Me = 7.00) vs. obesity (Me = 10.00) were statistically significantly different. The magnitude of the DMFT value was also significantly dependent on occupational status (*p* = 0.009). Those who were retired or receiving a pension had the highest DMFT value (Me = 10.50), and this value was significantly different from the DMFT size determined for the other groups of respondents. Education also influenced the DMFT index value; a statistically significant differentiation was obtained between those with primary/vocational education (Me = 7.00) vs. higher education (Me = 10.00) (Table 6).

### 3.2. Criteria for Calculating the DMFT and Their Impact on PDI, CDI, and OHHPI Values

This study also attempted to assess the influence of the diagnostic criteria for estimating the DMFT on the values of the preventive diet (PDI), cariogenic diet (CDI), and oral health hygiene and prevention (OHHPI) indices (Table 7). There was no significant effect of the analysed questions included in the DMFT on the values of the studied indices.

It was observed that the occurrence of carious cavities was diagnosed less frequently for those with a higher PDI index value (4.27 vs. 4.08). As the number of teeth involved in carious lesions increased, a decrease in PDI values was observed. In the group of subjects with caries as the reason for tooth extraction and among the study participants with no carious fillings, diet had a higher preventive potential (4.12 vs. 4.09, respectively).

When asked about the number of teeth extracted due to caries and the number of teeth in which carious fillings were restored, it was observed that with five teeth extracted or restored, the median preventive diet index was relatively high at 4.35 vs. 4.14, respectively. It can be assumed that the deterioration of the dental condition had a positive effect on the change in dietary habits.

In the course of the study, it was observed that the values of the cariogenic diet index did not vary significantly according to the DMFT criteria (medians were between 0.64 and 1.97). It was observed that the lowest values of the CDI index were characterised by subjects in whom the number of teeth extracted due to caries and the number of teeth in which carious cavities were restored was four teeth—0.64 and 0.87, respectively. In the case of subjects in whom carious cavities were observed during the dental examination, the value of the cariogenic diet index increased. As the number of teeth involved in caries or extracted because of caries increased, the study participants had a lower consumption of products contributing to tooth damage.

For the OHHPI, the median values also did not differ significantly according to the DMFT criteria and ranged between 4.50 and 5.50. Those with carious fillings had a higher OHHPI value. It was observed that those without carious lesions and those with five or more observed carious teeth had the highest oral hygiene status (OHHPI 5.06–5.50). In the case of the first group, a high level of oral hygiene explains the absence of carious lesions, whereas in the second group, increased attention to daily hygiene and the use of measures showing a prophylactic effect (chewing gum, use of fluoride preparations) may be a consequence of the fear of losing occupied teeth. Similar observations were made when assessing the number of teeth in which carious cavities were restored or the number of teeth extracted as a consequence of the development of caries.

### 3.3. Influence of Selected Dietary Habits on the Values of the PDI, CDI, OHHPI, and DMFT Indices

Correlations were determined between the prophylactic and procarious diet indices, the oral hygiene and prophylaxis index, and the dental caries index and selected dietary habits with a potential impact on the development of dental caries (Table 8).

A positive correlation was found between the length of intervals between meals and the DMFT index sum value (r = 0.23). Another significant correlation was the one describing the effect of the use of health-promoting sweeteners on the value of the preventive diet index (PDI) (r = 0.18). Study participants who knew and used sugar substitutes were more likely to consume preventive foods in the context of developing dental caries. In addition, a positive weak correlation was shown between the sweetening of hot drinks and the DMFT index value (r = 0.19). Those who sweetened hot drinks with a minimum of two teaspoons of sugar had a higher dental caries index. A negative correlation was observed between the use of health-promoting sweeteners and the DMFT index value (r = −0.25). Respondents who used health-promoting sweeteners were characterised by a lower total dental caries index (Table 8). In the present study, there was no statistically significant correlation between the selected dietary factors, oral health hygiene, and preventive activities. Similar observations were made for the CDI index.

A correlation was also established between the caries index value and the other indices assessed, namely the prophylactic and prokaryogenic diet index and the oral hygiene and prevention index. Only a statistically significant correlation occurred between the dental caries index and the index of oral hygiene and prevention (OHHPI). The correlation coefficient was −0.123, which indicates a weak negative correlation, i.e., that as the quality of the respondents’ oral hygiene maintenance and prevention knowledge increased, the number of teeth affected by caries decreased (Table 9).

## 4. Discussion

One of the main factors resulting from diet that influences the incidence of caries disease appears to be an abnormal BMI. Obesity and caries share a common modifiable factor that may increase the incidence of their development—diet and lifestyle. In the present study, there was no significant effect of the BMI value on the PDI, CDI, and OHHPI values, but a significant effect was observed for the DMFT value. The DMFT value was shown to be significantly higher in the obese group than in the normal-weight group. The consequence may be inappropriate eating habits, which contribute not only to weight gain but also to poor oral hygiene and an increase in the incidence of carious lesions. The results of Tschammler et al. [40] suggest that erosive tooth wear and caries experience in primary and permanent teeth were significantly increased in children with obesity compared to normal-weight children. The authors showed that behaviours contributing to obesity, poor toothbrushing habits, and the consumption of cariogenic beverages were identified as significant caries risk factors. Li et al. [41] in a study of 18-year-old adolescents in Hong Kong showed that males were significantly more likely to consume cereals, milk, and meat compared to females. Women, on the other hand, were more likely to report a higher consumption of sweets. A dental examination showed that 60.9 percent of the participants had dental caries. The percentage of participants with caries was significantly higher among women than among men. Participants in the study who consumed sweets at least once a day were significantly more likely to have caries than participants who declared consuming this group of products less than once a day. In addition, a significant association was found between oral health hygiene and prevention parameters and body weight. A higher DMFT value was associated with a higher likelihood of being overweight and obese. The DMFT value was also associated with WHR—a one-unit increase in the DMFT value was associated with a 12% increase in WHR. Similar observations were made in the present study. Indeed, it was shown that as the BMI value increased, the DMFT value increased. A study by Karki et al. [42] attempted to assess the impact of BMI values on the severity of carious lesions. The study involved 1135 Nepalese school children aged 5–6 years and 12–15 years. It was shown that untreated dental caries was prevalent in children with both low and high BMI values. In addition, the most intense caries process was diagnosed in children with infrequent tooth brushing habits and who frequently consumed sweet bakery products, sweets or candies, and tea with sugar. In a study that Cazzolla et al. conducted among children from Albania and Italy, attention was drawn to the importance of diet in dental diseases such as dental caries. It was associated with nutritional deficiencies and excesses of protein, vitamin A, vitamin D, and B vitamins as well as iron and calcium [43].

In a study of South Korean adults, it was noted that overweight and obesity had a direct association with the incidence of advanced dental caries, regardless of the other variables assessed (gender, age, lifestyle, and systemic disease information). The positive association between a high BMI vale and the incidence of advanced dental caries was most pronounced in the elderly and female population [44]. A meta-analysis by Kirthig et al. found significant effects of selected socio-demographic factors on the incidence of dental caries in children, e.g., a low level of maternal education, single or unemployed mothers, a low household income, and parental occupational status [45]. Karki et al. 2018 also conducted an assessment of the oral health status of Nepalese children and attempted to find an association between dental caries and sociodemographic factors. It was observed that children from smaller towns had a poorer oral health status than those living in urban areas. The effect of gender on oral health status was also shown [46].

These results are similar to those presented in the present study, suggesting that abnormal BMI values and poorer education significantly affect dental status by increasing the frequency and intensity of caries. It is also worth noting the correlation between the DMFT and OHHPI index values. The present study showed that subjects with a lower OHHPI index value had a higher caries DMFT index value. The reason for these poor oral hygiene habits may be, as in the case of BMI, socioeconomic status and the associated lack of awareness of oral hygiene.

The present study also observed an increase in caries intensity with the age of the subjects. Older people are at higher risk of developing caries due to frequent co-morbidities and medication. In 2015–2016, an oral health survey was carried out among 500 elderly people over 65 years of age in the city of Wrocław. The study showed that 100% of the participants had caries. Moreover, the DMFT rate among those surveyed aged 65 years and over, on average, was 27.5, and 80% of the elderly had had a tooth extraction due to caries complications [47]. A meta-analysis by Zhang et al. found that older adults, smokers, people with lower socio-economic status, those with poorer oral hygiene, and those with more exposed root surfaces are at higher risk of having caries [48]. A study by Kossioni et al. analysed the oral health status of the elderly in Greece. The study showed that approximately 60% of the surveyed individuals over 65 years of age had at least one carious cavity. The study also confirmed that older patients often have a higher risk of tooth decay due to ageing enamel and reduced saliva production [49]. It is also worth noting a comparative analysis of research findings conducted by Werdiningsih et al. In their literature review, they focused on the role of oral hygiene, smoking, and diet in the development of dental caries in the elderly. The results suggested that a diet high in sugars and sweets was associated with a higher risk of dental caries in older patients. The study also pointed to the problem of dry mouth. A lack of saliva and attempts to relieve symptoms by sucking on sugary sweets contributes to caries. It was recommended that older people should limit their intake of sweets and choose healthy, nutritious meals to maintain healthy teeth [50].

In this study, a relationship was observed between a more frequent consumption of products that had a potentially preventive effect on oral health and gender. “Preventive” products included fruits, vegetables, unsweetened dairy products, and whole-grain cereal products, among others. The World Health Organisation (WHO) suggests that reducing the intake of high-energy, processed foods high in fat and carbohydrates in favour of a greater consumption of fruits, vegetables, legumes, whole grains, and nuts is an important strategy for preventing diseases that can be caused by poor diet. These demands appear to be justified, especially given the correlation between overweight, obesity, and dental caries. A different observation was made in the study by Li et al. [41], where it was observed that the participants in the study did not follow the WHO recommendations. More than 25% of the participants claimed to consume vegetables only once a day. More than 65 and 84% of the participants consumed fruit and dairy products less than once a day. Comparing the data obtained with the values of the DMFT index, it was observed that in the group of participants characterised by a lower intake of dairy products, the DMFT took lower values. An inverse relationship was observed for the consumption of sweets, cereal products, fruit, and vegetables. The group that declared their more frequent consumption had higher DMFT values. Nevertheless, these relationships were not statistically significant.

This study also showed a correlation between the length of meal intervals and the value of the dental caries DMFT index. As the length of the interval between meals increased, its value increased. The reason for this may be the amount and pH of saliva produced, which is responsible for cleaning the interdental spaces and thus improving oral hygiene [49]. Similar relationships were shown in a 4-year study by Bjørndal et al. among 239 participants. The study assessed the eating habits and oral health of adults. The results showed that long gaps between meals were associated with a higher risk of caries [51]. Huang et al. conducted a study on dietary and oral health assessment among 571 children and their mothers from Vietnam. Among the children, a high sweet intake and the prevalence of overweight or obesity in one in four children were found. The prevalence and severity of dental caries increased with the age of the child. The authors of the study indicated that long gaps between meals and a frequent consumption of snacks, especially sugary ones, drinking fizzy drinks, and a low frequency of tooth brushing were associated with a higher risk of dental caries among children [52]. Among the conclusions drawn from the study, the effect of the use of sugar substitutes on lowering the tooth decay intensity and the correlation between the use of health-promoting sugar substitutes and a more frequent consumption of preventive foods were still mentioned (PDI). A study by Janket et al. in 2019 examined the effects of xylitol on oral health, proving that sugar substitutes can reduce the risk of tooth decay and can also act as an effective tool in preventing tooth damage [53]. Similar observations can also be made on the basis of a meta-analysis of 30 studies examining the effectiveness of sugar substitutes in the prevention of caries disease. The authors emphasise that xylitol should be part of an overall strategy to reduce and prevent dental caries. Doctors recommending xylitol as a means of preventing tooth decay should consider the dose and frequency of use of the product. Indeed, a consumption of less than 3.44 g/day has not shown any benefit in terms of caries prevention [54].

Oral health knowledge is crucial for health-related behaviours [55]. With the continuous development of dentistry, a wide range of oral hygiene products are available on the market [56]. Toothbrushes and toothpaste are the most commonly chosen products used for oral hygiene. However, guidelines indicate that other oral hygiene aids should also be used. Routine dental check-ups, frequency of brushing, and proper dietary habits, as well as a lower sugar intake, flossing, and other interdental cleaning techniques are essential to maintaining good oral health [57]. In the present study, adequate oral hygiene was shown to reduce the incidence of carious lesions (r = −0.123). A study by Moradi et al. (2019) among 2000 individuals aged 15–40 years from Iran showed that oral hygiene procedures such as tooth brushing, mouth rinsing, and flossing positively affect oral health, contributing to lower DMFT values [58]. Oral hygiene and its impact on the DMFT index value was also studied by Milona et al. (2021). The study involved 264 children aged 15 years old from north-western Poland. The participants completed a questionnaire on age, gender, frequency of dental visits, dietary habits, and oral hygiene. Based on the data obtained, the decayed missing and filled teeth (DMFT) index was calculated. It was shown that the DMFT rate was negatively correlated with tooth brushing after the last meal and daily flossing. In addition, Milona et al. showed that tooth brushing at least twice a day did not have a significant effect on the reduction in DMFT index values [59]. The occurrence of an association between oral hygiene activities and the DMFT index was assessed in a group of 34 nursing students in Turkey. The use of dental floss, mouthwash, and regular brushing twice a day significantly reduced the occurrence of carious lesions in the the study participants [60].

This study was carried out among adults and assessed dental health in relation to dietary habits and knowledge of hygiene practices. Many studies published to date refer mainly to children or the elderly, whereas this study focused on working adults. In addition, three survey instruments were created in this study: the cariogenic diet index (CDI), preventive diet index (PDI), and oral health hygiene and prevention index, which can be used by other researchers.

## 5. Conclusions

In this study, dietary choices influenced the incidence and intensity of dental caries. However, a healthy diet in order to prevent excess weight seemed to be more important than the frequency of consumption of specific food groups, as the index values of the prophylactic and procarious diets did not have a significant effect on the sum of the dental caries index. Limiting sugar and organic acid intake, eating a healthy, balanced diet rich in fibre, and avoiding frequent snacking between meals can help to maintain oral health and reduce the risk of caries. In addition, good oral hygiene, regular brushing, flossing, providing adequate fluoride, and regular visits to the dentist are extremely important to maintaining dental health and preventing tooth decay. The above research points to the need for systemic solutions aimed at oral health education involving all sections of society.

## Figures and Tables

**Table 1:** The general characteristics of studied group.

Socio-Demographic Characteristics	Total	
	N = 148	[%]
**Gender**		
Woman	92	62 16
Man	56	37.84
**Age (years)**		
18–34	41	27.70
35–59	86	58.11
60–75	21	14.19
**Place of residence**		
Village	25	16.89
City < 500,000 inhabitants	113	76.35
City ≥ 500,000 inhabitants	10	6.76
**Occupational situation**		
Student	15	10.14
Worker	107	72.30
Pensioner	22	14.86
Unemployed	4	2.70
**Education**		
Primary/vocational	11	7.43
Secondary	62	41.89
Higher	75	50.68
**Body Mass Index (BMI) (kg/m^2^)**		
<18.5	0	0
18.5–24.9	76	51.35
25.0–29.9	51	34.46
≥30.0	21	14.19

**Table 2:** Questions composing the preventive diet index (PDI) and the cariogenic diet index (CDI) [33].

Cariogenic Diet Index (CDI)	Preventive Diet Index (PDI)
Content of the question	
How often do you consume fruits?	How often do you consume sticky products such as toffee-type sweets, fudge, cookies, crackers, crisps?
How often do you consume vegetables?	How often do you consume acidic products such as citrus fruits, lemon, vinegar, pickled vegetables, pickled vegetables, vinaigrette-type sauces?
How often do you consume fibre-rich foods such as whole-grain bread, bran, mountain cereal, groats, whole-grain pasta, raw fruit or vegetable salads?	How often do you consume sweets such as candies, cakes, pastries, chocolate bars, muesli bars, other confectionery?
How often do you consume dairy products (milk and its derivatives, cheese, natural yoghurt)?	How often do you drink sweetened beverages such as Coca-Cola, Pepsi, Sprite, Fanta, orangeade, lemonade?
How often do you eat hard cheeses such as parmesan, pecorino, gouda, emmenthaler, cheddar, sapphire, ruby?	How often do you drink energy drinks such as Monster, 2 KC, Black Horse, Red Bull, Burn, Shot and others?
How often do you consume products that are sources of arginine (an amino acid) such as sunflower seeds, pumpkin seeds, squash, nuts, coconut, beans, soya, watermelon, tuna?	How often do you drink fruit juices?

**Table 3:** Recommended indicators for frequency of food intake in the KomPAN questionnaire [34].

Frequency Categories	Frequency Categories	Frequency Categories
Never	1	0
1–3 times a month	2	0.06
Once a week	3	0.14
Several times a week	4	0.5
Once a day	5	1
Several times a day	6	2

**Table 4:** Classification of dietary index scores and indices assessed in the nutrition and hygiene questionnaire.

Index	% Score	Range
Low	<33% max number of points	0–3.9
Medium	>33.1% <66% max number of points	4–7.9
High	>66.1% max number of points	8–12

**Table 5:** Oral health hygiene and prevention index—questions and scores [36,37].

Questions	How Often Do You Brush Your Teeth?		Do You Use Toothpaste Containing Fluoride?		Do You Use Any Other Methods of Oral Hygiene Than Brushing Your Teeth?		If So, Which Ones?		Do You Consider Chewing Gum to Be Part of Your Oral Hygiene?		How Often Do You Use Chewing Gum?	
**Answers and assigned points**	After every meal	2.5	Yes	1	Yes	1	Flossing	1	Yes	1	Never	0
	Twice a day	2									1–3 times a month	0.06
	Once a day	1.5	No	0			Tooth irrigation	1			Once a week	0.14
	Once every two days	1			No	0			No	0	Several times a week	0.5
	Once every three days	0.5	Don’t know	0.5			Tooth rinsing	1			Once a day	1
	Less than once every three days	0									Several times a day	2
							Other	1				

**Table 6:** Influence of selected socio-demographic factors and BMI on values of assessed indicators.

Factors		n	PDI				CDI				OHHPI				DMFT			
			Me	Q1	Q3	*p*	Me	Q1	Q3	*p*	Me	Q1	Q3	*p*	Me	Q1	Q3	*p*
**Age**	18–34	41	3.53	2.00	6.12	0.510	1.27	1.0	2.29	0.331	4.50	3.50	5.64	0.091	7.00 b	5.00	10.00	<0.001
	35–59	86	4.17	3.00	5.53		1.24	0.75	2.04		5.03	4.06	6.00		8.00 b	7.00	10.00	
	60–75	21	4.14	3.00	4.70		1.20	0.66	1.80		4.50	3.50	5.00		10.00 a	9.00	12.00	
**Gender**	Woman	92	4.48 a	3.14	6.00	0.007	1.21	0.71	2.01	0.308	5.20	4.00	5.82	1.000	8.00	6.00	10.00	0.130
	Man	56	3.21 b	2.11	4.70		1.36	0.82	2.13		5.00	3.50	5.82		9.00	6.50	11.00	
**BMI**	Valid	76	4.06	2.7	5.50	0.472	1.72	0.92	2.40	0.723	5.00	6.50	6.10	0.633	7.00 b	6.00	10.00	<0.001
	Overweight	51	3.78	2.57	5.10		1.21	0.67	1.90		5.00	4.06	6.00		9.00 ab	6.00	11.00	
	Obesity	21	4.70	2.81	6.10		1.20	0.68	1.30		5.00	3.50	5.10		10.00 a	8.00	11.00	
**Place of residence**	Village	25	4.03	2.56	6.00	0.712	1.20	0.69	2.10	0.964	5.00	3.50	5.50	0.587	7.00	6.00	10.00	0.189
	City < 500,000 inhabitants	111	4.06	2.81	5.10		1.27	0.79	2.00		5.00	4.00	6.00		8.00	7.00	10.00	
	City ≥ 500,000 inhabitants	12	4.59	2.72	7.56		1.48	1.14	1.62		4.31	2.50	5.60		6.00	2.00	7.00	
**Education**	Primary/vocational	11	4.62	4.00	5.14	0.353	0.81	0.53	1.67	0.306	4.50	3.14	5.14	0.272	10.0 a	8.00	12.00	0.004
	Secondary	62	3.74	2.59	5.14		1.42	0.83	2.06		5.00	3.50	5.56		9.00 ab	7.00	11.00	
	Higher	75	4.06	2.67	5.56		1.21	0.72	2.07		5.00	4.00	6.06		7.00 b	6.00	10.00	
**Occupational** **situation**	Student	15	4.12	2.59	7.56	0.591	1.43	0.83	3.02	0.341	4.50	3.06	6.06	0.286	6.00 b	2.00	7.00	0.009
	Worker	107	4.90	2.62	5.53		1.23	0.76	2.00		5.00	4.00	6.00		8.00 b	6.00	10.00	
	Pensioner	22	4.07	2.87	4.70		1.20	0.66	2.09		4.75	3.50	5.50		10.50 a	9.00	12.00	
	Unemployed	4	5.17	4.39	5.85		1.94	1.06	2.47		5.78	4.75	6.28		6.50 b	5.50	9.50	

**Me**—median of the variables in each group; **Q1:Q3**—values for the 25th and 75th quartiles in a given group; **a, b**—statistically significant differences; ***p***—probability in Kruskal–Wallis’ test or in Mann–Whitney’s U test; **PDI**—preventive diet index; **CDI**—cariogenic diet index; **OHHPI**—oral health hygiene and prevention index; **DMFT**—decayed, missing, and filled teeth index.

**Table 7:** Influence of selected oral hygiene factors on the values of the assessed indicators.

Factors		n	PDI				CDI				OHHPI			
			Me	Q1	Q3	*p*	Me	Q1	Q3	*p*	Me	Q1	Q3	*p*
**The presence of carious cavities**	yes	116	4.08	2.58	5.50	0.669	1.27	0.75	2.03	0.898	5.00	3.53	5.56	0.341
	no	32	4.27	3.03	5.04		1.27	0.75	2.24		5.03	3.82	6.03	
**Number of teeth affected by caries**	0	30	4.06	3.14	5.06	0.109	1.27	0.66	2.34	0.493	5.06	4.00	6.06	0.282
	1	45	4.56	3.50	5.56		1.27	0.79	2.09		5.00	4.06	5.60	
	2	24	4.09	3.10	6.08		1.50	0.93	2.01		4.50	3.07	5.57	
	3	17	4.01	2.09	4.70		1.21	0.81	1.70		4.56	3.06	5.06	
	4	13	2.78	1.45	5.00		1.01	0.59	1.21		4.50	4.00	5.14	
	5	17	3.03	2.17	4.20		1.27	0.75	2.04		5.50	4.00	6.06	
**Have teeth been extracted due to caries?**	yes	87	4.12	3.00	5.50	0.589	1.27	0.69	2.04	0.513	5.00	4.00	5.64	0.812
	no	61	4.03	2.56	5.50		1.27	0.81	2.22		5.00	3.50	6.06	
**Number of teeth extracted due to caries**	0	61	4.03	2.56	5.50	0.294	1.27	0.81	2.22	0.460	5.00	3.50	6.06	0.330
	1	23	4.14	2.34	6.00		1.27	1.02	1.71		5.56	3.50	6.06	
	2	17	5.00	4.00	5.64		1.40	1.08	1.67		5.06	5.00	6.50	
	3	11	3.67	2.64	4.12		1.27	0.75	2.04		4.50	3.06	5.50	
	4	6	2.82	1.48	4.53		0.64	0.25	1.08		5.07	4.50	5.50	
	5	30	4.35	3.00	5.14		1.34	0.53	2.14		5.00	4.00	5.06	
**The presence of carious fillings**	yes	144	4.05	2.69	5.50	0.645	1.27	0.75	2.05	0.795	5.00	3.60	5.82	0.767
	no	4	4.09	3.46	6.16		1.13	0.51	2.45		4.53	3.78	5.53	
**Number of teeth in which decay has been restored**	0	4	4.09	3.46	6.16	0.687	1.13	0.51	2.24	0.210	4.53	3.78	5.53	0.956
	1	4	3.51	3.14	5.32		1.97	0.77	3.65		4.81	3.60	6.03	
	2	7	3.70	2.59	8.14		1.60	1.06	3.02		4.50	3.00	5.50	
	3	5	4.56	3.28	4.62		1.04	0.44	2.09		5.06	4.14	5.56	
	4	21	3.64	1.81	4.70		0.87	0.56	1.42		4.50	4.00	5.50	
	5	107	4.14	2.81	5.64		1.27	0.81	2.04		5.00	3.50	6.06	

**Me**—median of the variables in each group; **Q1:Q3**—values for the 25th and 75th quartiles in a given group; ***p***—probability in Kruskal–Wallis’ test or in Mann–Whitney’s U test; **PDI**—preventive diet index; **CDI**—cariogenic diet index; **OHHPI**—oral health hygiene and prevention index.

**Table 8:** Spearman’s correlation coefficient for selected questions from the nutrition and hygiene questionnaire regarding dietary habits and the indicators assessed.

Factors	Spearman’s Correlation Coefficient			
	PDI	CDI	OHHPI	DMFT
Length of breaks between meals	0.00	−0.04	−0.02	0.23
Frequency of snacking during the day	0.03	0.06	−0.02	0.01
Frequency of snacking at night	0.03	−0.05	−0.01	−0.10
Sweetening of hot drinks	0.07	−0.01	−0.03	0.19
Use of health-promoting sweeteners	0.18	0.00	−0.04	−0.25

**PDI**—preventive diet index; **CDI**—cariogenic diet index; **OHHPI**—oral health hygiene and prevention index; **DMFT**—decayed, missing, and filled teeth index.

**Table 9:** Kendall’s tau correlation coefficient between DMFT and the other indicators assessed.

Variable	Kendall’s Tau Correlation Coefficient		
	PDI	CDI	OHHPI
DMFT	0.071	−0.073	−0.123

**PDI**—preventive diet index; **CDI**—cariogenic diet index; **OHHPI**—oral health hygiene and prevention index; **DMFT**—decayed, missing, and filled teeth index.

## Data Availability

The data presented in this study are available on request from the corresponding author.
